# Investigating Pregnancy and Its Complications Using Circulating Cell-Free RNA in Women's Blood During Gestation

**DOI:** 10.3389/fped.2020.605219

**Published:** 2020-12-14

**Authors:** Mira N. Moufarrej, Ronald J. Wong, Gary M. Shaw, David K. Stevenson, Stephen R. Quake

**Affiliations:** ^1^Departments of Bioengineering and Applied Physics, Stanford University, and Chan Zuckerberg Biohub, Stanford, CA, United States; ^2^Department of Pediatrics, Stanford University School of Medicine, Stanford, CA, United States

**Keywords:** transcriptome, cell-free RNA, preeclampsia, prediction, preterm birth, IUGR

## Abstract

In recent years, there have been major advances in the application of non-invasive techniques to predict pregnancy-related complications, for example by measuring cell-free RNA (cfRNA) in maternal blood. In contrast to cell-free DNA (cfDNA), which is already in clinical use to diagnose fetal aneuploidy, circulating RNA levels can correspond with tissue-specific gene expression and provide a snapshot of prenatal health across gestation. Here, we review the physiologic origins of cfRNA and its novel applications and corresponding challenges to monitor fetal and maternal health and predict pregnancy-related complications.

## Introduction

**Figure 1:**
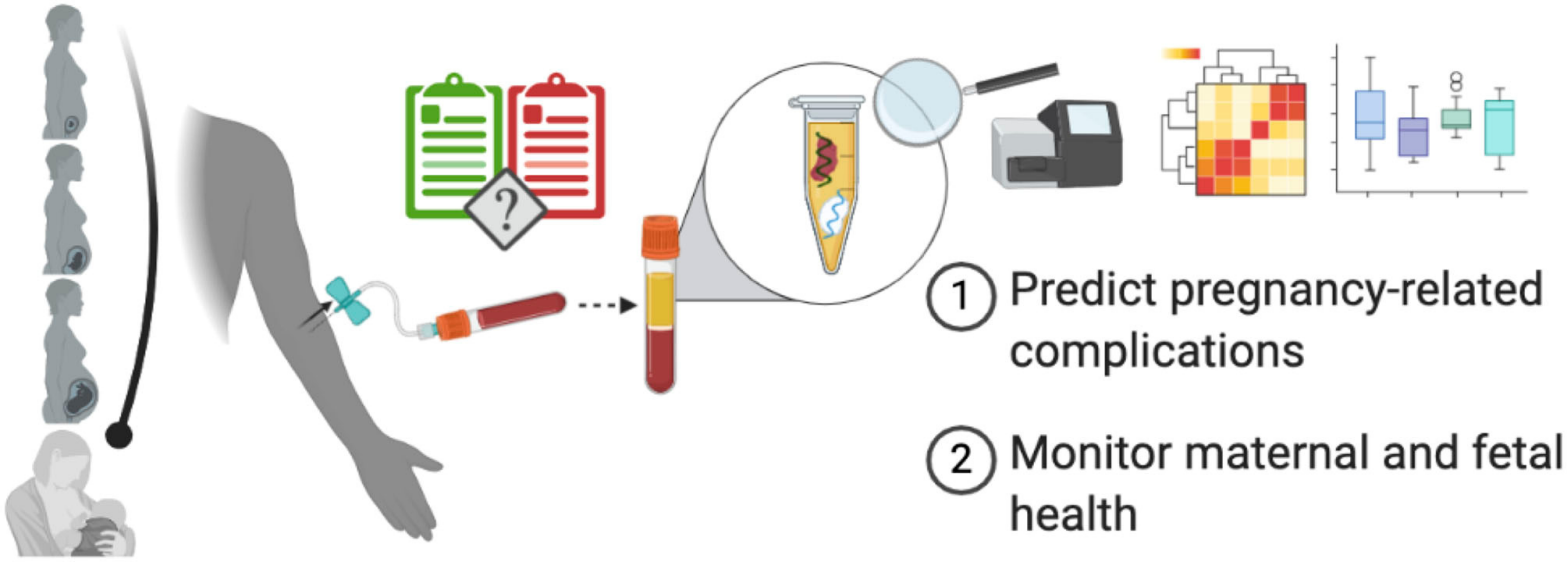
Using cfRNA to monitor pregnancy and predict pregnancy-related complications. From conception until delivery, cfRNA isolated from maternal plasma can provide a snapshot of fetal and maternal health and may even predict pregnancy-related complications prior to their clinical onset, thereby helping guide clinical practice. Created with BioRender.com.

For years, ultrasound has been the primary tool used to monitor fetal health allowing for the direct measurement of fetal shape, size, and some aspects of fetal physiology, all of which are important for development. It would be very useful to have a companion tool, which enables measurement of the molecular details of development, like the gene expression profile of the fetus over time. Liquid biopsies that rely on a simple maternal blood sample offer a possible avenue to realize this goal. Indeed, the measurement of circulating cell-free DNA (cfDNA) to screen for fetal aneuploidy as first demonstrated 12 years ago (1) has already seen wide-spread clinical adoption (2). More recently, in the past 5 years, we have witnessed unprecedented leaps in the applications of this technology to monitor prenatal health following its extension to measurements of circulating RNA in whole blood or cell-free RNA (cfRNA) in plasma. Today, circulating and cfRNA measurements have been applied to predict and characterize pregnancy-related complications like spontaneous preterm birth (PTB) (3), preeclampsia (4), and intrauterine growth restriction (IUGR) (5). In contrast to cfDNA levels, cfRNA levels of specific genes change during gestation in predictable ways that map closely with their placental and fetal gene expression (3, 4, 6–9). These timed patterns provide a snapshot of prenatal health across gestation and can be leveraged to predict pregnancy-related complications months before clinical diagnoses can be made and perhaps even used to monitor fetal health. Here, we review the physiology of cfRNA, its novel applications, and its challenges as they relate to fetal and maternal health (Figure 1).

## Physiologic Origins of Cell-Free Nucleic ACIDS During Pregnancy

Circulating RNA in pregnant women was first identified by Mandel and Metais in 1948 (10)—long before the invention of reverse-transcription quantitative polymerase chain reaction (RT-qPCR) or next generation sequencing (NGS). Without the necessary means to interrogate the nucleic acids they identified, this discovery laid dormant until the 2000s when researchers leveraged RT-qPCR and NGS to answer five key questions about the physiologic origins of prenatal cfRNA. Specifically, they asked who (mom or fetus), what (RNA types), where (tissues), why (cellular activity), and when (clearance) contribute to prenatal cfRNA levels and in what fractions.

Several groups have now confirmed through orthogonal methods that both the fetus and mother contribute to measured cfRNA levels (3, 4, 6–9, 11, 12). Their fractional cfRNA contributions can be estimated by detecting exomic single-nucleotide variations (SNVs) at sites where either mother or child, but not both, is heterozygous. Although the mother and child may share both alleles across many genes (i.e., both heterozygous or both homozygous), we can still obtain reasonable, informative estimates of fractional contributions. Late in the first trimester, fetal-specific contributions comprise <1% of the total cfRNA measured (6). Later across gestation, fetal-specific contributions conservatively increase from <4% before 18 weeks of gestation to about 15% after 24 weeks (6, 9).

Furthermore, cfRNA from many types of RNA, including circular and non-coding, can be measured in the cf-transcriptome throughout pregnancy (6). As Koh et al. highlight, most fetal cfRNA is coding (i.e., mRNA), whereas about 15% appears to be non-coding (i.e., no open reading frame, long intergenic non-coding, or antisense that survives DNAse treatment). The fractional contribution of microRNA across gestation as compared with other RNA types remains unknown—owing to the unique technical challenges of isolating, amplifying, and sequencing both small and long RNA molecules.

To address where in the body prenatal cfRNA originates from, researchers used available body atlases (13) to identify tissue-specific genes that are also detected prenatally. Defining what constitutes “tissue-specific” expression remains an evolving question as more atlases become available (14–18). In general, it is defined as when expression of a gene in a single tissue can be measured exclusively or several fold (typically 2–20) above that from all other tissues. Using this definition, many genes detected prenatally originate from the placenta (9, 11, 19, 20); however, other tissues and body systems also contribute measurably like fetal tissues and the maternal immune system (3, 4, 7, 21, 22).

What specific cellular process—death, active signaling, or both—gives rise to cfRNA in circulation remains an open question (20, 23–25). In general, cfRNA is both protected from degradation and remarkably stable after blood collection (26). Protection can occur in the form of encapsulation in extracellular vesicles or apoptotic bodies suggesting both active secretion and cell death as possibilities, and most evidence available today remains indirect. Based on prenatal cfDNA and placental research, the majority of cfDNA arises from placental cell turnover, which occurs constantly (27, 28). Therefore, prenatal cfRNA may be released by the same placental cell turnover. Extending this idea to pregnancy-related complications, it is possible that placental-specific complications could result in higher cell death and consequently increased cfRNA release, although this remains an active area of research. Indeed, the connection between apoptosis and proliferation for both cfDNA as reviewed elsewhere (29), and by extension, cfRNA, points to apoptotic nucleic acid release as a means to understand both dying and living, proliferating cells. Recent convincing evidence outside the area of pregnancy also points to the possibility of active secretion (30). In longitudinal studies of cancer patients who underwent bone marrow ablation, Ibarra et al. found that cf-mRNA captured active transcriptional activity. Specifically, they observed that bone marrow cell type-specific cfRNA level changes preceded parallel changes in the circulating cell count of the same cell type. These findings correspond with expected behavior during active secretion and exactly contrast behavior during cell death, where cfRNA level changes should follow those for cell counts.

Finally, there remains the question of cfRNA clearance or how long do RNA molecules remain in circulation following their release. In general, for healthy pregnancies, we expect all cell-free nucleic acids to clear within hours of delivery (3, 6, 8, 31). More specifically, it is known that certain placental cf-mRNAs have a half-life of about 14 min following delivery for healthy pregnancies (19), and most pregnancy-related cfRNA has been observed to clear from circulation within hours of delivery (6, 8, 11, 32). In contrast, women with preeclampsia (31) or women who underwent elective first-trimester surgical termination (33) both experienced delayed clearance of fetal cfDNA or cfRNA, respectively. Generally, the effect of pregnancy-related complications on cfRNA clearance remains an open question; however, we can hypothesize that placental-specific complications (like placenta accreta) would also result in the delayed clearance of placental-specific cfRNA molecules post-delivery when compared with healthy pregnancies.

## Longitudinal cfRNA Changes Over Uncomplicated, Full-Term Gestation

Intriguingly, several groups have shown that cfRNA levels for specific pregnancy-related genes and circular RNAs change over gestation in predictable patterns (4, 6). Late in gestation, some of these changes map closely with placental gene expression (9) or fetal organ function (7). They may also reflect maternal immune system dynamics (8). Furthermore, prenatal changes in only nine placental genes predict gestational age (GA) for healthy pregnancies with comparable accuracy to ultrasound, providing a possible alternate method to accurately date pregnancies in the second and third trimesters (3). Importantly, most pregnancy-specific genes, typically those of placental or fetal organ origin, clear from circulation within 2 h of delivery (11, 32) with the exception of certain maternal immune genes that eventually return to measurable non-pregnant baseline levels (3, 8).

Longitudinal changes in gene expression follow three main trends: those that change continuously over pregnancy, those that change early in pregnancy and reach a plateau late in the second trimester, or, conversely, those that remain constant early in pregnancy only to change dramatically later in gestation (3, 4, 6, 8). In uncomplicated full-term pregnancies, the cfRNA profiles of placental, maternal immune, and fetal tissue-specific genes correlate with those of other genes specific to the same body system. Placental and fetal tissue-specific cfRNA levels also moderately correlate with each other (3). Despite successful measurement of fetal tissue-specific genes individually, their detection early in gestation can be variable, thereby impeding broad meaningful conclusions about fetal health and organ development. Overall, their predictive power has yet to be thoroughly explored.

## Understanding and Predicting Pregnancy-Related Complications Using Circulating RNA Levels

Until the last 2 years, research with the goal of predicting pregnancy-related complications has focused on measuring very few genes using RT-qPCR in small, single-hospital cohorts without external validation. These studies have been thoroughly reviewed elsewhere (20) and will not be our focus here. Recently, three groups have systematically identified (using RNA-sequencing, RNAseq) and separately validated (using RT-qPCR) changes in gene levels between matched cases and controls for spontaneous PTB (3), preeclampsia (4), and IUGR (5). The aforementioned studies all used samples from distinct collection sites for discovery and validation to increase generalizability. Nonetheless, each will require larger blinded validation prior to clinical adoption.

Prediction of spontaneous PTB has been reported in Black women at two sites in the USA, where women either presented with symptoms of early labor or experienced a prior spontaneous PTB in the discovery and validation cohorts, respectively. In validation, cfRNA levels for seven genes successfully identified women who delivered preterm up to 2 months in advance of delivery; however, both discovery and validation cohorts were small (3). Larger follow-up studies in ethnically diverse populations that include asymptomatic women will be required to understand the generality of these results.

Another study used cfRNA to characterize preeclampsia. They focused on women with early onset, defined as a diagnosis prior to 34 weeks of gestation and severe features. Blood was sampled at the time of diagnosis between 24 and 34 weeks of gestation. The cfRNA levels of 49 genes, of which 19 were specific to fetal or placental tissue, successfully identified women who experienced early-onset preeclampsia with an accuracy of 85–89% in a small, independent validation cohort (4). The ability of this test to predict preeclampsia prior to symptomatic onset remains unknown and will likely be crucial to understanding its clinical utility. Specifically, prediction of preeclampsia early in gestation may guide the prophylactic use of low-dose aspirin starting at or before 16 weeks of gestation, which has been shown to significantly reduce the risk of preterm preeclampsia (34).

Finally, Hannan et al. (5) focused on identifying pregnancies with severe IUGR using whole blood sampled at the time of diagnosis. In validation, they showed that two (EMP1 and PGM5) of the five genes identified during discovery significantly differed between women carrying significantly growth-restricted fetuses and women with uncomplicated pregnancies at the same GA. Like Munchel et al. (4), the ability of this test to identify pregnancy-related complications prior to symptomatic onset remains unknown. Here, the authors suggest that these genes could be used to monitor IUGR after its identification via ultrasound; however, what further clinical information these genes will provide if used as a monitoring tool remains to be determined.

Other studies have also reported on differences between early-onset preeclampsia (35), women with adverse pregnancy outcomes in general (36), or women at high risk of experiencing preeclampsia (37) as compared with uncomplicated full-term pregnancies; however, they either did not include results for a separate validation cohort (35, 36) or only included limited results. Tsang et al. (35) identified specific placental cell-type signatures using single-cell sequencing and then measured these signatures in maternal plasma. They found that the signature for extravillous trophoblasts was elevated at diagnosis for women with early-onset preeclampsia as compared with GA-matched controls. Separately, Del Vecchio et al. (36) investigated whether cfRNA measured prior to diagnosis could predict several pregnancy-related complications and identified five cfRNA molecules that could predict preeclampsia in the first term for a very small discovery cohort (five cases of preeclampsia). Without an external validation dataset, it is difficult to interpret the results of both of these small, but encouraging, studies. Finally, Srinivasan et al. (37) explored the utility of bivariate microRNA ratios to predict preeclampsia in high-risk women prior to diagnosis. They reported that certain microRNA ratios could distinguish between women who went on to experience preeclampsia and those who did not as early as 17 weeks into pregnancy; however, limited validation results were provided. Furthermore, as aforementioned, the ability of any test to predict preeclampsia prior to 16 weeks remains untested, but critical to guiding prophylactic aspirin use.

The field of prenatal cfRNA diagnostics has seen many exciting developments in the past 5 years; however, as noted by others previously (20), these results are still awaiting large-scale validation. Nonetheless, the inclusion of small, but independent, validation cohorts in three of these studies increases their likelihood of generalizability (38) and provides further systematic confirmation of the diagnostic value of measuring cfRNA levels.

## Experimental Challenges When Isolating Circulating RNA

As a short-lived molecule in its naked form, RNA can prove challenging to isolate. Although cfRNA specifically is known to be surprisingly stable in circulation, several experimental decisions may influence research conclusions including the sample type, collection tubes, and volumes as well as processing, extraction, and amplification protocols. Here, we will highlight current best practices to ensure high sample quality and review quality metrics that can be used to identify samples with unusually high levels of RNA degradation, DNA contamination, or ribosomal RNA. Generally, cfRNA isolated from plasma can be extracted and then prepared for RNAseq or RT-qPCR using some of the many commercially available kits as described below.

Circulating RNA can be isolated from whole blood, serum, or plasma—all of which require specific blood collection tubes. Tubes for whole blood collection like PAXgene, Qiagen, or Tempus RNA contain stabilizing agents that cause maternal cell lysis, thereby increasing background signals and obscuring the specific origin—cellular or cell-free—of any RNA measured. Similarly, coagulation of the blood sample required prior to the isolation of serum also leads to cell lysis resulting in the same obfuscation as first identified with circulating DNA (39). On the other hand, plasma isolation from whole blood requires tubes with reagents that inhibit coagulation and stabilize blood cells, thereby reducing the likelihood of cell lysis. Tubes used for plasma isolation should contain either EDTA, ACD, Streck cfDNA, or Streck cfRNA solutions, but not heparin as the latter has been shown to inhibit reverse transcription (40). In sum, plasma is the ideal sample type to identify pregnancy-specific cfRNA alone and minimize any background from maternal blood cell lysis.

A major concern when using plasma has been the recommendation to isolate plasma immediately after whole blood collection to ensure sample quality. Recent work by Munchel et al. (4) has shown that overnight shipping and storage had no adverse effect on measured pregnancy-associated signals for any common tube used for plasma isolation. Furthermore, they showed a strong cfRNA level agreement broadly (Pearson's *R* ≥ 0.7) and, more specifically, for prediction of early-onset pre-eclampsia across paired samples for which plasma was isolated 0 to 5 days post-collection of blood in Streck cfDNA tubes.

cfRNA can be successfully extracted and amplified from a minimum of 0.5 ml of plasma with strong agreement between technical replicates (30). In a direct comparison, Munchel et al. (4) recommended that 2–4 ml of plasma be used as starting material to minimize variability and maximize library complexity. Since this may not always be possible when using samples from biobanks, which may have limited quantities, findings by Ibarra et al. emphasize that even smaller initial volumes like 0.5 ml can be used.

Finally, extraction and amplification of sufficient cfRNA have persisted as challenges. Recent work has converged on several best practices. Like most RNA-based discovery work, cfRNA analysis can be separated into three parts—extraction, amplification, and downstream analyses.

Published work today relies on commercially available RNA extraction kits from Norgen Biotek Corp., Qiagen, or Thermo Fisher Scientific for example. Importantly, RNA extraction must be followed by DNA digestion as recently emphasized in a re-analysis (41, 42) of data produced by a novel technique, SILVER-seq, to isolate cfRNA from 3 μl of serum that may have also isolated cfDNA (43). Furthermore, quality control measures should be taken to ensure DNA digestion occurs as expected. Following digestion, cfRNA can be cleaned and concentrated using commercially available kits like those from Zymo Research to a final volume of 15 μl or less. The amount, size, and integrity of the isolated RNA can be confirmed using Agilent's RNA 6,000 Pico chip.

Following RNA extraction, samples may be prepared for sequencing or RT-qPCR. Library preparation for sequencing has converged on two key methods—total RNA sequencing with ribosomal RNA (rRNA) depletion (8) and exonic-focused (i.e., mRNA) sequencing via whole-exome enrichment (4, 30). The appropriate method depends on desired downstream analyses. Total RNA sequencing permits simultaneous measurements of many RNA types (circular, non-coding, or coding) and non-human sequences, some of which map to human bacteria and viruses (8); however, even following rRNA depletion, in our and another group's experience, typically 10–20% of all reads will still map to ribosomal sequences (3, 4) and only about 20–85% (mean of 35%) will map to coding regions. On the other hand, about 90% of reads from whole-exome enrichment and sequencing map to coding genes, thereby trading off a high exonic mapping efficiency with loss of potential information from non-coding RNA or non-human sequences (4, 8). Best practices for RT-qPCR have not changed in some time and similarly rely on commercially available kits that use TaqMan probes.

## Metrics to Quantify Sample Quality

Even with best practices, sample quality can still vary. Several quality metrics can be used to identify samples with unusually high levels of RNA degradation, DNA contamination, and/or ribosomal RNA (44). Any sample for which at least one of these three metrics deviates from expected values described below should be considered a quality outlier and excluded from further analyses. In our experience, we find that these metrics are well-calibrated and quickly flag water samples, a negative control, as quality outliers. Indeed, when we visualize the principal component analysis (PCA) of cfRNA data from all samples, we find that the samples flagged as outliers by these metrics cluster separately from most samples and typically drive the first or second principal component, thereby introducing unwanted technical bias. Removing these sample outliers from any downstream analysis circumvents the introduction of such bias.

For degraded input material like cfRNA, the RNA integrity number (RIN) may not be informative. Instead, to assess RNA degradation, researchers can leverage that ribonucleases present in human plasma exhibit 5′-3′ catalytic activity owing to poor 3′ poly(A) cleavage (45). Specifically, by counting and annotating identified exons from RNAseq data, researchers can determine the fraction of genes for which all assigned reads come from the 3′ exon (44). Based on our own work using total RNAseq for almost 700 samples, we expect sample quality such that around 25–40% (with a median of 28%) of all genes will contain reads from only the most 3′ exon. Samples for which this metric is above 40%, the 95th percentile, likely contain significantly degraded cfRNA and may have been mishandled prior to plasma isolation.

To assess DNA contamination, one can estimate the intron-to-exon read ratio by counting the number of reads that map exclusively to intronic as compared with exonic sequences (44). Here, we expect most samples to have a ratio of <1, indicating more exonic than intronic reads. This is not a perfect metric since non-coding RNA will inflate the ratio and can be mistaken for DNA contamination, but it remains useful as a rule of thumb. There are opportunities to develop more sophisticated informatic subtractions of known transcribed regions of the genome, which will add more accuracy to this metric. In our samples, we generally expect an intron-to-exon ratio of around 0.20–2.85 with a median ratio of 0.60 and consider any values >3, the 95th percentile estimated from nearly 700 cfRNA samples, to be extremely unusual and typically the result of very few exonic reads (i.e., a small denominator).

Finally, to estimate the rRNA fraction, researchers can count reads that map to known ribosomal sequences as compared with the total number of reads for a given sample. Here, we expect values of 10–20% for total RNAseq as previously noted and <10% for whole-exome enriched RNAseq (4, 8, 44).

## Computational Challenges When Interpreting Circulating RNA Measurements

Most computational methods for cfRNA data analyses mirror those used for any RNA-based application. RNAseq data pre-processing relies on tools to remove adapter sequences from reads (i.e., trimmomatic or cutadapt), map reads (i.e., STAR), remove PCR duplicates (i.e., Picard or samtools), and finally count the number of reads that map to any given gene (i.e., htseq-count, featureCount, or STAR). RT-qPCR analysis requires far fewer pre-processing steps, namely, just normalization as described in more detail below. Subsequently, to identify genes associated with pregnancy-related complications, researchers frequently use methods like differential expression (i.e., limma-voom, DESeq2, or EdgeR) or machine learning (i.e., sklearn). Despite the numerous pre-processing steps in common between cfRNA and general RNA analyses, there remain several important differences as well.

Unlike RNA measurements from specific tissues, cfRNA levels represent a mixture of RNA from many tissues and both mother and fetus for any given gene (6). Interpreting any specific gene result can be especially difficult and, for some measures, relies on the completeness of relevant atlases. The recent rising popularity of single-cell techniques underscores the importance of contextually defining tissue or cell “specificity.” For cfRNA present in circulation, identifying genes as cell-specific requires that they be unique in expression both for an individual tissue and across adult and fetal tissues. Importantly, special attention should be paid to the specificity of any RNA molecule in both adult and fetal tissues as it is possible that an otherwise “tissue-specific” RNA molecule in atlases that examined adult tissues alone could be expressed elsewhere in early development. Consequently, applying single-cell data from one organ to interpret plasma cfRNA as described previously (35) may be exciting but premature, as the specificity of cell-specific gene signatures in the context of the whole body prenatally remains unknown.

Furthermore, standard practice when analyzing cellular RNA may not apply as expected to cfRNA since it represents a mixture of RNA levels from across the body. One such example is the use of reference genes to normalize RNA levels across samples. When planning RT-qPCR experiments and choosing an appropriate number of reference genes, we point the reader to the MIQE guidelines (46). In the context of prenatal cfRNA, researchers should be especially cognizant that traditional reference gene levels like those for GAPDH may be variable across people and gestation, and one group has reported on more stable reference genes to use when studying pregnancy (47). Moreover, reference genes can be avoided by converting cycle thresholds to molecular counts with a standard curve (3). In all inquiries, initial plasma volumes should also be normalized for if they differ across samples.

Finally, cfRNA discovery work is also plagued by the same issues as any method that seeks to identify predictive signatures (48, 49). Building a classifier should pull exclusively from discovery data with validation data used only for testing as opposed to further feature selection or model training. In practice, applying such a rigid boundary can be tricky but necessary as validation metrics may otherwise prove overly positive. Concretely, building a model during discovery with a set of genes, but refining it in validation to only include a subset that showed significance again (5), falls into this paradigm. In these cases, although the individual gene changes hold, the aggregate reported statistics for the validation set are likely to be overly optimistic.

## Future Applications

Recent years have seen several proof-of-concept studies, which suggest that cfRNA can be a powerful diagnostic tool. However, owing to the study scale required to prospectively collect enough samples from women with pregnancy-related complications, all of the highlighted studies used at least some samples collected at onset of symptoms or at diagnosis. While encouraging, this present emphasis on prediction at diagnosis cannot replace the required work to show that cfRNA diagnostics can also anticipate complication onset and consequently allow for clinical intervention. To address this, large-scale, prospective studies hold promise as they can both confirm prediction at and prior to diagnosis.

In contrast to several studies so far that have focused on cf-mRNA, future applications may explore the predictive power of different types of RNA. Data from total RNA-sequencing like that from Koh et al. (6) have revealed that other RNA types, like circular RNA or non-coding RNA, can also change over gestation in predictable patterns. Furthermore, work like that by Srinivasan et al. (37) points to the potential utility of microRNAs to predict risk of preeclampsia. Altogether, novel methods that capture both small and long RNA molecules could provide an opportunity to directly compare the clinical utility of many RNA types to predict pregnancy-related complications.

Future applications may also benefit from incorporating measurements from other nucleic acids entirely like cfDNA or other molecules like proteins and metabolites as has recently proven informative for prostate cancer (50) and PTB prediction (51), respectively. One recent report that examined both cfDNA methylation and cfRNA measurements for pregnancy-related complications found that in a small discovery cohort, placental-specific cfDNA increased prior to the subsequent development of gestational diabetes and that other cfRNA changes preceded preeclampsia (36). Withstanding validation and further exploration, these studies point to the possibility of incorporating cfDNA, proteins, metabolites, and other molecules with cfRNA in future prenatal applications as recently highlighted by Paquette et al. (52).

Separate from the prediction of pregnancy-related complications, another goal is to monitor fetal health in real-time. Despite encouraging results, researchers have thus far only identified a small number of fetal genes in circulation and noted the variabilities of such detection early in gestation when the fetus contributes <4% of the total cfRNA measured. Creating signatures, which combine information from groups of co-regulated genes may prove key to developing fetal tissue-specific signatures that can be reliably measured across gestation.

Furthermore, extension of such work from correlative observations to causative inference remains a major challenge not only for cfRNA analyses but also for RNA discovery work broadly. Here, we can imagine applying both cutting-edge computational and experimental methods like the construction of generative models or the application of correlative cfRNA insights to refine experimental hypotheses. Nonetheless, such experimental or computational work must be informed by our knowledge about the physiology of cfRNA. Here, one important question remains unanswered: What biological process—active secretion, cell death, or both—produces prenatal cfRNA?

Step by step, researchers have answered many important questions in a field that is only two decades old. Excitingly, groups over the past few years have clearly shown that cfRNA is not only a rich data source but also one with diagnostic potential. Yet still, key questions must be addressed for cfRNA diagnostics to be clinically used and impact prenatal care.

## Author Contributions

MNM, RJW, GMS, DKS, and SRQ conceptualized this review. MNM wrote the original manuscript draft and created the overview figure. All authors revised the manuscript and approved it for publication.

## Conflict of Interest

SQ is a founder and shareholder of Mirvie, and Stanford University and the Chan Zuckerberg Biohub have filed patents based on the work of SQ and MM on the use of cfRNA in maternal and fetal health. The remaining authors declare that the research was conducted in the absence of any commercial or financial relationships that could be construed as a potential conflict of interest.
