# Corrigendum: Investigating Pregnancy and Its Complications Using Circulating Cell-Free RNA in Women's Blood During Gestation

**DOI:** 10.3389/fped.2021.680201

**Published:** 2021-04-12

**Authors:** Mira N. Moufarrej, Ronald J. Wong, Gary M. Shaw, David K. Stevenson, Stephen R. Quake

**Affiliations:** ^1^Departments of Bioengineering and Applied Physics, Stanford University, and Chan Zuckerberg Biohub, Stanford, CA, United States; ^2^Department of Pediatrics, Stanford University School of Medicine, Stanford, CA, United States

**Keywords:** transcriptome, cell-free RNA, preeclampsia, prediction, preterm birth, IUGR

In the original article, the **Conflict of Interest** statement was inadvertently incomplete. The statement should include:

“SQ is a founder and shareholder of Mirvie, and Stanford University and the Chan Zuckerberg Biohub have filed patents based on the work of SQ and MM on the use of cfRNA in maternal and fetal health.

The remaining authors declare that the research was conducted in the absence of any commercial or financial relationships that could be construed as a potential conflict of interest.”

The authors apologize for this error and state that this does not change the scientific conclusions of the article in any way. The original article has been updated.

